# Incentivizing Verifiable Privacy-Protection Mechanisms for Offline Crowdsensing Applications

**DOI:** 10.3390/s17092024

**Published:** 2017-09-04

**Authors:** Jiajun Sun, Ningzhong Liu

**Affiliations:** College of Computer Science and Technology, Nanjing University of Aeronautics and Astronautics, Nanjing 211106, China; jiajunsun@nuaa.edu.cn

**Keywords:** mobile crowdsensing, privacy protection, verifiable correctness, incentive mechanisms

## Abstract

Incentive mechanisms of crowdsensing have recently been intensively explored. Most of these mechanisms mainly focus on the standard economical goals like truthfulness and utility maximization. However, enormous privacy and security challenges need to be faced directly in real-life environments, such as cost privacies. In this paper, we investigate offline verifiable privacy-protection crowdsensing issues. We firstly present a general verifiable privacy-protection incentive mechanism for the offline homogeneous and heterogeneous sensing job model. In addition, we also propose a more complex verifiable privacy-protection incentive mechanism for the offline submodular sensing job model. The two mechanisms not only explore the private protection issues of users and platform, but also ensure the verifiable correctness of payments between platform and users. Finally, we demonstrate that the two mechanisms satisfy privacy-protection, verifiable correctness of payments and the same revenue as the generic one without privacy protection. Our experiments also validate that the two mechanisms are both scalable and efficient, and applicable for mobile devices in crowdsensing applications based on auctions, where the main incentive for the user is the remuneration.

## 1. Introduction

Recently, crowdsensing has received extensive attention since it can solve complex sensing issues by pervasive mobile devices worn by the ordinary users. For instance, BX Tracker [1] measuring human mobility and signal coverage based on GPS tracking in cellular networks, VTrack [2] having real-time and omnipresent traffic state information and NoiseTube [3] drawing noise maps. Although these crowdsensing applications have been developed, incentive mechanisms are indispensable for achieving good-quality services. Consequently, some researchers such as Singer et al. [4,5], separately propose auction mechanisms to incentivize extensive users to participate in crowdsensing applications so as to meet the previous service demands [4,5,6]. These novel mechanisms not only guarantee the truthful participation of users by adopting near-optimal prices of assignments for crowdsensing applications with a budget constraint, but also satisfy the incentive compatibility, budget feasibility, constant competitive ratio, thereby ensuring these mechanisms applicable to crowdsensing applications.

Despite their merits, payments’ verifiability and privacy issues as two critical human factors in crowdsensing applications have not been fully explored. A common hypothesis made in the above mechanisms is that the involved parties will follow the protocols honestly without the concern of their privacy. However, some users could behave selfishly to protect their cost privacy, sensing preferences’ privacy and identification privacy, thereby violating the hypothesis and making these well-designed mechanisms inefficient. On the other hand, the platform needs to keep the set of current winners secret to maximize his utility when facing the adversarial users. Thus, it is imperative to provide some measures to eliminate the privacy-leakage concerns of users and the platform so as to achieve good service quality.

In addition to the privacy issue, the payments’ verifiability issue is also a crucial human factor for the wide acceptance of the above crowdsensing applications. It is because that some controller of the platform (the crowdsensing organizer) may misbehave, e.g., provide false results or insert a fictitious bid and sensing preference so as to deceitfully pay users at a lower cost [7,8]. If the correctness of the payments from the platform is not well guaranteed, users will be reluctant to participate in crowdsensing applications. In practice, since it is an individual (e.g., a public servant) that administers a real-world platform in a large corporation, or in a government department, the possibility of incorrect operations from the platform exists in crowdsensing applications. For example, according to the estimation of the World Bank, about $200 billion per year results from incorrect exchanging hands for public sector procurement and close to $1.5 trillion resulted from the taint of incorrect operations to the procurement projects. Thus, how to deal with the payments’ verifiability is crucial for the success of crowdsensing applications.

Although both privacy and verifiability issues have been identified as two crucial human factors, many existing research works [9,10,11,12] tend to investigate them seperately. For instance, some privacy enhanced techniques [13,14] enable a user to hide his identity and sensing profile (i.e., cost and sensing preferences like locations), but some verifiable strategies, especially the non-truthfulness incentive strategies, may be hard to implement in the above truthful incentive mechanisms, since the platform needs to greedily select winners and compute the threshold payment based on the examination of a user’s sensing profile. However, the improvement of the verifiability needs to reveal more information, thereby reducing privacy of users and platform. Therefore, how to simultaneously address privacy and verifiability problems will become particularly challenging in crowdsensing applications [15,16].

While the work in [17] investigated the online verifiable privacy-protection crowdsensing issue only for the heterogeneous sensing job model, the offline verifiable privacy-protection crowdsensing issues are more complex and challenging since the payment correctness of the platform is not verified by participatory users who have left the mechanism. On the contrary, in this paper, we investigate the offline verifiable privacy-protection crowdsensing issues. To address the above-mentioned challenges, we present a first step towards building a crowdsensing system in which users can verify the payments from the platform without revealing any additional information by applying the order preserving encryption scheme (OPES) [18]. Our approach is to enable users to verify the payments with the help of an auction issuer (AI): the AI chooses winners and greedily computes the threshold payment based on encrypted user’s sensing profiles. Since these encrypted sensing profiles are order-preserving, the threshold payment is the same as the one produced by the platform, thereby solving the verifiability without reducing privacy of users and platform. Specifically speaking, we first introduce three incentive mechanisms for crowdsensing applications with homogeneous sensing jobs, heterogeneous sensing jobs and submodular sensing jobs (to be elaborated later). Then, we propose a general verifiable privacy-protection incentive mechanism for homogeneous sensing jobs and heterogeneous sensing jobs and that for submodular sensing jobs respectively, as shown in Table 1, where H-PVA (Heterogeneous-user based Privacy-preservation Verifiable Auction) is an online mechanism based on heterogeneous sensing jobs [17]. The two mechanisms are implemented by introducing the oblivious transfer (OT), the bulletin board, and the timed lapse cryptography services (TLC), satisfying the above-illustrated three desirable properties: the non-repudiation by the platform and users, verifiable correctness, and secrecy [12]. Finally, analysis shows that our verifiable privacy-protection mechanisms achieve the similar results as the generic mechanism without privacy preservation and verification.

The rest of the paper is organized as follows. In Section 2, we briefly discuss the related work and motivation. In Section 3, we present our relative models and our design goal. In Section 4, we introduce novel incentive mechanisms for crowdsensing applications with the budget constraint. Based on these mechanisms, in Section 5, we design two privacy-preserving verifiable incentive mechanisms satisfying the above three desirable properties, followed by the security analysis and performance evaluation in Section 6 and Section 7. Finally, we draw our conclusions in Section 8.

## 2. Background and Related Work

Privacy-protection mechanisms have received extensive attentions in crowdsensing applications [20,21,22,23,24,25]. The work of [26] applied the *k*-anonymity method to protect users’ privacy by hiding a user’s location under a cloak of k−1 other users. The works of [27,28] use the temporal and spatial cloaking techniques to protect users’ privacy by blinding the participant’s location of a cloaked area at a specific time for satisfying the privacy requirements. The work of [29] employs a novel spatiotemporal probabilistic k-anonymity for blurring mechanisms based on tessellation and protecting users’ privacy. The work in [22,30,31,32] considered the privacy protection based on the privacy regulation in crowdsensing applications. Furthermore, the work in [31,32] mainly investigate how participatory users fulfill the jobs for the platform while without revealing their identity. Different from the above mechanisms, the work of [21] explores the differential privacy for protecting the privacy of each user’s bid against the other honest-but-curious users. The work of [9,23] introduces the OT [33] for protecting the users’ privacy. The work of [22] integrates the incentive, data aggregation and perturbation for providing reliable data, and compensating their costs of sensing and privacy leakage. The work of [34] introducing the optimal quality of information for protecting users’ privacy under the condition of without knowing the trajectories of participants However, all of these works do not consider the verifiability of user’s inputs and outputs.

Besides, verifiability of the payment is coexistence with the above privacy protection for an incentive mechanism design. The verifiability of payments have been extensively explored in traditional auction mechanisms. For instance, the work of [35,36,37] forms a proxy-OT based circuit for validating the payment of the platform. The work of [7,38] keeps the platform knowing users’ bids for performing a timed lapse cryptography service until the auction is closed so that it is impossible to rig their bids for participatory users after bidding. However, all these works do not apply for realistic crowdsensing applications, since they neglect the effect of a large of participants in crowdsensing applications. More recently, a timed commitment encryption method is introduced to enhance the level of the payment correctness from the platform. For example, the work of [11,12,17,39,40] apply the timed commitment to address the verifiable correctness issue from different aspects. While, these works are not feasible to real crowdsensing applications with the limited budget.

In this paper, to solve the above challenges, we introduce the bulletin board, OT, and TLC to guarantee the privacy and verifiability for the offline crowdsensing applications without sacrificing the platform’s utility and truthfulness.

## 3. System Model and Problem Formulation

In this section, we expound our system model, auction model, adversarial model, and the bulletin board, which will be applied to our verifiable privacy-protection incentive mechanisms. Further, the detailed goal is introduced.

### 3.1. System Model

**Figure 1:**
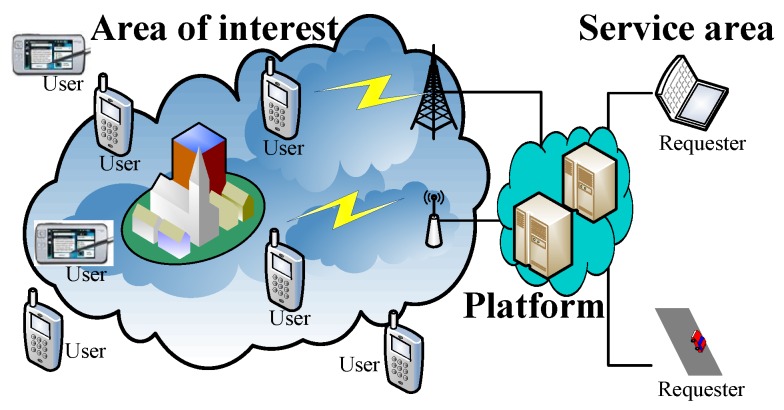
Our crowdsensing system model.

We take the following system model for crowdsensing applications into account, illustrated in Figure 1. The system is composed of a crowdsensing application platform, which consists of multiple sensing servers in the cloud, and many users, whose mobile devices are connected to the cloud by wireless networks, e.g., cellular networks including GSM/3G/4G or WiFi connections. The requester posts a crowdsensing task with a budget B>0 to the platform. There are *m* available assignments in each task. Receiving the task, the platform publicizes a crowdsensing campaign towards the area of interest (AoI), aiming at finding some users to maximize the number of assignments performed efficiently. Assuming that a set of users U={1,2,⋯,n} in the AoI is interested in the campaign. In this paper, with respect to the model of sensing jobs completed by all users, we discuss the following three sensing job models proposed in [4] for the crowdsensing campaign:

**Homogeneous sensing job model:** Both each sensing job assignment and the limit of the number of assignments completed by each user are the same. Meanwhile, each user can complete only a single assignment.

**Heterogeneous sensing job model:** Each sensing job assignment is the same, but the limit of the number of assignments completed by each user is different. It means that different users can complete different number of assignments.

**Submodular sensing job model:** Each sensing job assignment is different, and each user *i* can do a subset $\Gamma_{i}$ of assignments Γ.

If the campaign is oriented to users with the homogeneous sensing job model and the heterogeneous sensing job model, receiving the campaign, each user *i* synchronously submits his sensing profile $P_{i}=\left(b_{i},l_{i}\right)$, where $b_{i}$ is obtained based on a true cost $c_{i}$ for performing a single assignment and $l_{i}$ is a limit for the number of assignments he is willing to work on. This means that if he is a winner, he will be allocated at most $l_{i}$ assignments, thereby the payment for each assignment will exceed $b_{i}$. In this case, the sensing job model is the heterogeneous sensing job model, which indicates that different users can complete different number of assignments. When $l_{i}=1$, the sensing job model is reduced to the homogeneous sensing job model, which indicates that each user can complete only a single assignment, i.e., the homogeneous sensing job model can be defined as the subset of the heterogeneous model with $l_{i}=1$.

If the campaign is oriented to users with the submodular sensing job model, receiving the campaign, each user *i* synchronously submits his sensing profile $P_{i}=\left(b_{i},\Gamma_{i}\right)$, where $b_{i}$ is obtained based on a true cost $c_{i}$ for performing the sensing service with his assignments’ set $\Gamma_{i}$, i.e., $\Gamma_{i}\subseteq \Gamma =\left\{\tau_{1},\tau_{2},\cdots ,\tau_{m}\right\}$. We assume that $l_{i}$ or $\Gamma_{i}$ is fixed. Furthermore, under the budget constraint *B*, the platform, when presented with the sensing profiles of all users, must decide a subset of users to select, and how much payment to pay to each selected user. Our goal is to make incentive mechanisms to achieve non-repudiation by users and platform, verification, and secrecy without sacrificing the above standard economic goal such as utility maximization, truthfulness.

In practice, the above interactive process can be modeled as a sealed-bid auction between users and the platform, in which an AI is attached between a crowdsensing platform and a set of participatory users. The AI is semi-honest (passive or curious), and only checks the platform randomly. Simultaneously the platform also needs to maintain a bulletin board. All public information must carry appropriate digital signatures if they need to be posted to the bulletin board so that their originators can be identified.

### 3.2. Adversarial Model

In the auction process with the budget constraint, the platform is supposed to know only the set of current winners, and their sensing profiles. Each user *i* only learns whether he is the winner, and he is paid if he is a winner. He does not know anything about others’ profiles except for the very limited implicit information in the payment from the platform.

Assume that the platform and users are semi-honest adversaries in our mechanisms, and collusion of bidders and platform does not exist. That is, the platform is interested in inferring each user’s private information no matter he is a winner or not. Users try to infer others’ sensing profiles to maximize their own utilities. Besides, the platform and users can also collude with each other. According to the above auction model, we give the analysis of the privacy in our framework below.

**Definition** **1.** Given all the communication strings C and its output of the auction Output during the auction, an adversary’s advantage over the privacy information $\zeta_{i}$ of user i is defined as $Adv_{\zeta_{i}}=P_{r}\left[\zeta_{i}|C,Output\right]-P_{r}\left[\zeta_{i}|Output\right]$, where $P_{r}\left[\zeta_{i}\right]$ is the probability that a correct $\zeta_{i}$ is inferred. In this paper, $\zeta_{i}$ can be the bid or sensing services $\Gamma_{i}$ of user i.

**Definition** **2.** *A privacy-protection scheme satisfies k-anonymity, if a user cannot be identified by the sensitive information with probability higher than 1/k* [26].

In this paper, our security goal is to achieve a scheme such that the advantage is of a negligible function of the security parameter or *k*-anonymity is guaranteed.

### 3.3. Problem Formulation

According to the above sensing job model, we need to consider two cases. One case is when sensing job model is the homogeneous sensing job model or the heterogeneous sensing job model. In the two models, a mechanism M=(f,p) designed has an allocation function $f:R_{+}^{n}\to Z_{+}^{\left[n\right]}$ and a payment function $p:R_{+}^{n}\to R_{+}^{n}$. The function *f* denotes the mapping from a set of *n* bids to an assignments’ allocation for a chosen subset of users. In particular, in the homogeneous sensing job model, if user *i* is chosen, $f_{i}=1$. The function *p* returns a payments’ vector $\left(p_{1},\cdots ,p_{n}\right)$ to users. The goal of the platform aims to maximizing the number of assignments under the given budget *B*, i.e., $\max\sum_{i\in S}f_{i}$, subject to $\sum_{i\in S}f_{i}p_{i}\leq B$, $\forall i,f_{i}\leq l_{i}$. In particular, when $l_{i}=1$, the above results are also applicable to the homogeneous sensing job model.

The other case is when sensing job model is the submodular sensing job model. a mechanism M=(f,p) designed also has an allocation function $f:R_{+}^{n}\to 2^{\left[n\right]}$ and a payment function $p:R_{+}^{n}\to R_{+}^{n}$. However, the allocation function is different from the previous one. The function *f* here is a indicator function that returns 1 if user *i* is allocated and 0 otherwise. The utility of user *i* is $p_{i}-c_{i}$ if it is chosen, i.e., i∈S, 0 otherwise. The payment function *p* is the same to the previous one. The goal of the platform can be denoted as maximizing the value from the selected users’ services under the given budget *B*, i.e., maxV(S), subject to $\sum_{i\in S}p_{i}\leq B$, where V(S) satisfies the monotone submodularity.

However, the above goals also come with many privacy and security issues, such as users’ sensing profiles, due to the two following reasons. One is that users are reluctant to reveal all their private information to the platform and other users. The other is that both the winners and the platform have the ability of verifying the payment resulted from our mechanisms. Thus, our mechanisms not only satisfy the standard economic goals such as truthfulness, individual rationality, utility maximization, but also fulfil the following three desirable ones: non-repudiation by users and platform, secrecy and verifiable correctness, illustrated in [17].

## 4. Incentive Mechanisms for CrowdSensing Applications

In this section, to explicitly present our proposed mechanisms, we introduce three incentive mechanisms for crowdsensing applications with homogeneous sensing jobs, heterogeneous sensing jobs, submodular sensing jobs respectively. In essence, the incentive mechanisms for crowdsensing applications require the truthfulness, computationally effectiveness, budget feasibility and approximation (see [6]). Singer et al. present these mechanisms meeting the four conditions well.

To better understand the following incentive mechanisms, let us see the following familiar example. Given a budget constraint *B* and subsets U={1,2,⋯,n} of some ground set, where each user *i* corresponds to a subset of the ground set and a associated cost $c_{i}$ find a users’ subset *S* which maximizes $|\cup_{i\in S}\left\{i\right\}|$ under the given budget. This is a typical coverage problem, called submodular sensing job model here, in which each user’s value depends on the identity of the sensing data set it holds. When each user’s value only depends on the cardinality of the sensing data set, rather than the identity of the sensing data set it holds, it means that different users can complete different number of sensing data, thereby simplifying the submodular sensing job model to heterogeneous sensing job model. Furthermore, if each user only completes a single sensing data assignment, the heterogeneous sensing job model will become a homogeneous sensing job model. For the simplicity of presentation, we first introduce the incentive mechanism with homogeneous sensing job model.

### 4.1. Incentive Mechanism with Homogeneous Sensing Jobs

For crowdsensing applications with homogeneous sensing jobs, consider the above-mentioned allocation function *f*: Sorting the *n* bids reported by *n* users so that $b_{1}\leq b_{2}\leq \cdots \leq b_{n}$, and finding the largest *k* so that $b_{k}\leq B/k$. That is, *k* is the location at which the hyperbola B/k intersects the curve of the increasing costs.The set allocated here should be {1,2,⋯,k}. That is, winners’ set S={1,2,⋯,k}. Obviously, this is a monotone allocation function: a user can be not excluded when his bid is decreased. In [4], the authors design the following incentive mechanism for crowdsensing applications with homogeneous sensing jobs and show the mechanism satisfies the above four conditions.

More formally, firstly, sorting the users’ bids: satisfying $b_{1}\leq b_{2}\leq \cdots \leq b_{n}$. Then finding the largest integer *k* satisfying $b_{k}\leq B/k$. Finally, determine the set of allocated users to be S={1,2,⋯,k}, and provide same payment $p_{i}=\min\left\{B/k,b_{k+1}\right\}$.

### 4.2. Incentive Mechanism with Heterogeneous Sensing Jobs

For crowdsensing applications with heterogeneous sensing jobs, the authors of [5] present the following mechanism for determining near-optimal prices of jobs for crowdsensing applications with heterogeneous sensing jobs. Their mechanism is illustrated as follows: Firstly, sort the users’ bids: satisfying $b_{1}\leq b_{2}\leq \cdots \leq b_{n}$. Then find the largest integer *k* satisfying $b_{i}\leq B/\sum_{j\leq i}f_{j}$. Finally, determine the set of allocated users is given as S={1,2,⋯,k}, and provide the same payment $\min\{B/\sum_{j\leq i}f_{j},b_{i+1}/l_{i+1}\}$ for completing a sensing job.

Obviously, the homogeneous user model can be viewed as a special case of the heterogeneous user model, i.e., $l_{i}=1$ for each user *i*.

### 4.3. Incentive Mechanism with Submodular Sensing Jobs

For crowdsensing applications with submodular sensing jobs, the authors of [4,6,41] apply the proportional share allocation rule proposed in [4] to address the extensive user participation issue for crowdsensing applications, which is composed of two phases: the winner selection phase and the payment determination phase [19]. We first introduce definition of the submodular utility function.

**Definition** **3** **(Submodular** **Function).** Let N be a finite set, a function U : $2^{\Omega }\to R$ is submodular if U(S∪{i})−U(S)≥U(T∪{i})−U(T),∀S⊆T⊆Ω, where R is the set of reals.

From the above Definition 3, we can know the utility function *U* is submodular and derive the following sorting according to the increasing marginal contributions relative to their bids from users’ set to find the largest *k* satisfying $b_{k}\leq U_{k}B/U\left(S\cup k\right)$.

(1) $$U_{1}/b_{1}\geq U_{2}/b_{2}\geq \cdots \geq U_{\left|U\right|}/b_{\left|U\right|},$$

where $U_{k}$ denotes $U_{k|S_{k-1}}$ ($=U\left(S_{k-1}\cup \left\{k\right\}\right)-U\left(S_{k-1}\right)$), $S_{k}=\left\{1,2,\cdots ,k\right\}$, and $S_{0}=\emptyset$. To calculate the payment of each user, we can sort the users in U\{i} as follows:

(2) $$U_{i_{1}}\left(T_{0}\right)/b_{i_{1}}\geq U_{i_{2}}\left(T_{1}\right)/b_{i_{2}}\geq \cdots \geq U_{i_{n-1}}\left(T_{n-2}\right)/b_{i_{n-1}},$$

The marginal value of user *i* at the position *j* is $BU_{i(j)}\left(T_{j-1}\right)/U\left(T_{j}\right)$. Assume that $k^{\prime }$ to be the position of the last user $i_{j}\in U\backslash \left\{i\right\}$, such that $b_{i_{j}}\leq U_{i(j)}\left(T_{j-1}\right)B/U\left(T\right)$. To ensure the truthfulness, each winner should be given the payment of the critical value. This indicates that user *i* can not win the auction if it reports higher than this critical value. More details are given in Algorithm 1, where $b_{i(j)}=U_{i(j)}\left(T_{j-1}\right)b_{i_{j}}/U_{i_{j}}\left(T_{j-1}\right)$ and $\eta_{i(j)}=U_{i(j)}\left(T_{j-1}\right)B/U\left(T_{j-1}\cup \left\{i\right\}\right)$.
**Algorithm 1** An Auction Mechanism for Submodular Sensing Jobs Under the Budget Constraint**Input:** User set U, the budget constraint B.**Output:** The set of winners *S*.1:// Phase 1: Winner selection2:S←∅; $i\leftarrow \arg\max_{j\in U}U_{j}\left(S\right)/b_{j}$;3:**while** $U_{i}\left(S\right)/b_{i}\geq U\left(S\cup i\right)/B$ **do**4: S←S∪i;5: $i\leftarrow \arg\max_{j\in U\backslash S}\left(U_{j}\left(S\right)/b_{j}\right)$;6:**end while**7:// Phase 2: Payment determination8:**for** each user i∈U **do**9: $p_{i}\leftarrow 0$;10:**end for**11:**for** each user i∈S **do**12: $U^{{}^{\prime }}\leftarrow U\backslash \left\{i\right\}$; T←∅;13: **repeat**14:  $i_{j}\leftarrow \arg\max_{j\in U^{{}^{\prime }}\backslash T}\left(U_{j}\left(T\right)/b_{j}\right)$;15:  $p_{i}\leftarrow \max\left\{p_{i},\min\left\{b_{i(j)},\eta_{i(j)}\right\}\right\}$;16:  $T_{j-1}\leftarrow T$; $T\leftarrow T\cup \{i_{j}\}$;17: **until** $b_{i_{j}}>U_{i(j)}\left(T_{j-1}\right)B/U\left(T\right)$18:**end for**19:**return** (*S*, *p*)

However, although the above three mechanisms under the given budget constraint are so promising, we also need to address the previous-mentioned challenges. To this end, in the following section, we will explore two verifiable privacy-protection incentive mechanisms for homogeneous and heterogeneous sensing jobs, and submodular sensing jobs.

## 5. Design Details

In this section, we first introduce basic cryptographic schemes. Then we apply the schemes to design our verifiable privacy-protection mechanism for homogeneous, heterogeneous jobs and submodular jobs respectively.

### 5.1. Basic Cryptographic Schemes

We firstly construct the time-lapse and OT cryptography services for making users’ sensing profile secret. Then we give the blind digital signature for achieving the goal of non-repudiation by the platform and users, and the computation of marginal utility and set union for making platform’ current winners’ set secret. In the following details, we apply the bulletin board and the parameter α to ensure verifiability of payments and the payment correctness.

#### 5.1.1. Time-Lapse and OT Cryptography Services

In the following mechanisms we apply timed commitments on sensing profiles of all users until the auction closes. Cryptographic methods, as presented in [42] can be used to implement the timed-commitments. Considering the computation efficiency reasons, we choose a time lapse cryptography (TLC) service from [43], which makes it possible to use commitments with the classical hiding and binding properties. Besides, it prevents users from refusing to reveal committed sensing profiles and also preventing the platform from dropping received commitments, claiming not to have been able to reveal the committed sensing profiles. In our mechanisms, an auction issuer (AI), acting as the TLC service provider, publishes a public key of a non-malleable encryption scheme, and sends the corresponding private key only when the auction closes. Whenever timed commitments on sensing profiles are applied, it means that a user encrypts her sensing profile by applying the AI-generated public encryption key. Besides, receiving the corresponding private key, the platform can know the encrypted sensing profile.

OT is a secret exchange way between two parties, e.g., users and a platform. Each user only know one of *n* secrets, and the platform does not know which of the *n* secrets has been known. An efficient 1-out-of-*z* OT of integers [17,44] will be used in our works.

#### 5.1.2. Blind Digital Signature

In our work, each user is a signer who is introduced only to keep the confidentiality of its the following transformed bid and sensing subset of assignments to the platform as well as other users. Considering the security, not all digital signature schemes can be used [10]. To these goals, we apply the Nyberg-Rueppel signature scheme [45] (see Algorithm 2). Notably, we do not need the signer to verify the authenticity of them, and on the other hand the platform can obtain their transformed bids and sensing preference selections from all signers. Let $sign_{i}\left(m\right)$ denote as the message *m*’ signature from the user *i* and the value of the signature is an integer. Note that the signature scheme requires the message to be an integer, therefore, we need to apply $sign(\left\lfloor 10^{k}m\right\rfloor )$ for the input *m* if *m* is not an integer like the bid, where *k* can be appropriately chosen to preserve the rank from {3,4,⋯} and ψ(x) denotes the output of the signature scheme. At the same time, we remove the signature by using $10^{-k}sign^{-1}\left(c_{m}\right)$, where $c_{m}$ is obtained by the signature $c_{m}=sign_{i}\left(m\right)$. According to [10], the deviation for the roundness of the signature is negligible. Thus, our assumption is reasonable.
**Algorithm 2** Blind Nyberg-Rueppel Signature1:Initialize a prime number *p*, a prime factor *q* of p−1, and an element $g\in Z_{p}^{*}$ with order *q*;2:The signer selects $\tilde{k}\in Z_{p}$ and sends $\tilde{r}=g^{\tilde{k}}$(mod *p*) to signee;3:The signee randomly chooses $\alpha \in Z_{q}$, $\beta \in Z_{q}^{*}$, computes $r=mg^{\alpha }$(mod *p*) and $\tilde{m}=r\beta^{-1}$(mod *q*) until $\tilde{m}\in Z_{q}^{*}$. Then, he sends $\tilde{m}$ to the signer;4:The signer computes $\tilde{s}=\tilde{m}x+\tilde{k}$(mod *q*) and sends $\tilde{s}$ to the signee;5:The signee computes $\tilde{s}=\tilde{s}\beta +\alpha$(mod *q*), and the pair (r,s) is the the signature for *m*;6:Check whether $m=g^{-s}y^{r}r$(mod *q*) to verify the correctness.

#### 5.1.3. Marginal Utility Computation

Besides the above losers’ sensing preferences, the current winner set *S* produced by the platform, should be also kept secret to all users. In such problems, how to compute the marginal utility without knowing *S* is challenging. We address it by introducing multivariate polynomial evaluation protocol (MPEP), in which the polynomial are computed without revealing any $x_{i}$ input of various users as follows: $f\left(\vec{x}\right)=\sum_{k=1}^{m}\left(c_{k}\prod_{i=1}^{n}x_{i}^{d_{i,k}}\right)$, where there is a group of open *m* powers for each user and *m* coefficients to any participant as well as the attackers. We compute the marginal utility by assuming that there are *m* sensing data points and *n* mobile users. Then we have *m*-dimensional vector $C_{S}$ indicating whether *m* sensing data points are included in currently chosen sets *S*, where $c_{k,S}=1-\prod_{j=1}^{n}\left(1-c_{j,k,S}\right)$. If *k*-th data point is in user *j*’s subset $\Gamma_{j}$ of assignments and user *j* is in *S*, $c_{k,S}=1$, and 0 otherwise. Since each user knows whether it belongs to *S*, each winning user’s marginal utility can be evaluated via one aggregator MPEP with the help of *n* users and only user *i* receives the result by applying the above MPEP equation. Finally, the user *i* can divide his bid $b_{i}$ to the result to compute the marginal-utility-per-bid value $\omega_{i}$. The detailed expression is given as follows:

(3) $$\begin{array}{cc} \omega_{i} & =\frac{1}{b_{i}}\left(\sum_{j=1}^{m}c_{j,S\cup \{i\}}-\sum_{j=1}^{m}c_{j,S}\right)\\ & =\frac{1}{b_{i}}\left(\sum_{k=1}^{m}\left(1-\prod_{j=1}^{n}(1-c_{j,k,S\cup \{i\}}\right))-\sum_{k=1}^{m}\left(1-\prod_{j=1}^{n}(1-c_{j,k,S}\right))\right). \end{array}$$

#### 5.1.4. Privacy Preservation Set Union Computation for Platform

Since the current winner set *S* is required to be kept secret to all users, for the platform, how to compute the set union without leaking its privacy, i.e., the current winner set *S* is a challenging issue. In the paper, we address it by using Paillier cryptosystem [46] to the set union computation [47]. The detailed description is illustrated in Algorithm 3.
**Algorithm 3** Privacy-Preserving set Union Computation1:Initialize system parameter: two same-length prime numbers *p*,*q*, public keys n=pq, $g\in Z_{n^{2}}^{*}$, private key λ=(p−1)(q−1), $\mu =\lambda^{-1}$mod *n*;2:The platform computes the polynomial $f_{A}$ and sends the encrypted $E_{p}\left(f_{S}\right)$ to the user $u_{i}$;3:Upon receiving $E_{p}\left(f_{S}\right)$, the user $u_{i}$ chooses a random value *r* (choose uniformly) and computes a tuple $\left(E_{p}\left(f_{S}\left(\tau \right)\times \tau \times r\right),E_{p}\left(f_{S}\left(\tau \right)\times r\right)\right)$ for each assignment value τ∈S. He randomly permutes all of the tuples and sends them to the platform;4:For each tuple $\left(E_{p}\left(x\right),E_{p}\left(y\right)\right)$, the platform decrypts *x* and *y*. If both values are 0, then the platform continues to next tuple. Otherwise, the platform finds a good with the value $x\times y^{-1}$ and adds it to the output set; As such, the marginal utility of the user $u_{i}$ can be obtained.

### 5.2. Design Privacy-Preserving Details for Homogeneous and Heterogeneous Jobs

#### 5.2.1. Initialization

The platform sends the following information to the AI: the deadline *T*, and its task identifier TID, and the timed-lapse encryption key TPK applied by all users in commitments. If the AI accepts them, he will set the probability of the auditions from users as α so that $\alpha \geq p_{max}/\left(F+p_{max}\right)$, where $p_{max}$ and *F* are the maximal payment and fine paid from the platform respectively, and sends signed α and signed auction details to the platform. If it is accepted by the platform, it will be posted to the bulletin board. $\beta =\left\{\beta_{1},\beta_{2},\cdots ,\beta_{z}\right\}$ and $\chi =\left\{\chi_{1},\chi_{2},\cdots ,\chi_{v}\right\}$ respectively are denoted as a set of possible bids and a set of possible limits of the number of assignments, where $\beta_{1}<\beta_{2}<\cdots <\beta_{z}$ and $\chi_{1}<\chi_{2}<\cdots <\chi_{v}$ hold, and requires each user *i*’s bid $b_{i}\in \beta$ and the limit of the number of assignments $l_{i}\in \chi$. The AI maps each bid value $\beta_{i}$ and limits value $\chi_{i}$ respectively to $\gamma_{i}$ and $\tau_{i}$, while keeping the rank, i.e., $\gamma_{1}<\gamma_{2}<\cdots <\gamma_{n}$ holds and $\tau_{1}<\tau_{2}<\cdots <\tau_{n}$. Similarly, users’ bids and limits are transformed by using the OPES for preserving their ranks. Assume that the above AI can bootstrap the crowdsensing market application and all of the previous data signatured by the platform $sign_{p}$ can be posted on the bulletin board.

#### 5.2.2. Commitment

**Figure 2:**
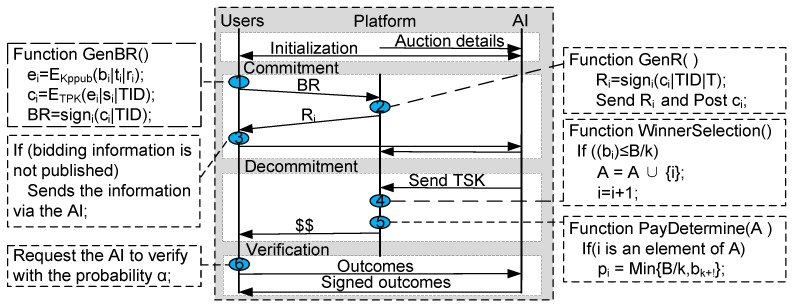
Our privacy-preserving verifiable framework for homogeneous and heterogeneous jobs.

Choosing a limit $l_{i}$ of the number of assignments and a bid $b_{i}$ to form his sensing profile, user *i* interacts with the AI. User *i* receives ${\tilde{b}}_{i}=\gamma_{x}$ and his limit ${\tilde{l}}_{i}=\tau_{x}$ according to [17], which are the rank-preserving encrypted values of $\beta_{x}$ and $\chi_{x}$ respectively, thereby forming his encrypted sensing profile. Then user *i* encrypts the encrypted profile as $e_{i}=E_{K_{ppub}}({\tilde{b}}_{i}|{\tilde{l}}_{i}\left|r_{i}\right)$ by applying the platform’s Paillier encryption key $K_{ppub}$ and a randomly selected values $r_{i}$. User *i* makes a commitment $c_{i}=E_{TPK}(e_{i}|s_{i}\left|TID\right)$, where $s_{i}$ is a randomly generated bit string for the correctness proof. Finally, the user will sign this commitment, and send a bidding request $BR=(sign_{i}\left(c_{i}|TID\right))$ to platform, if used, before time *T* (see Figure 2, step ①). The platform returns a signed receipt $R_{i}=sign_{p}(c_{i}\left|TID|T\right)$ (see Figure 2, step ②). At time *T*, the platform will post all the received true commitments $c_{1},c_{2},\cdots ,c_{n}$ on the bulletin board.

Note that the secondary encryption is applied to hide the encrypted bids and the random strings, thereby keeps anyone from learning any knowledge of the data prior to time *T*. Particularly, neither the AI nor the platform has any meaningful information.

Furthermore, between time *T* and T+1, for any user who has a receipt for a bid which is not posted (see Figure 2, step ③), his non-inclusion can be appealed and resorted to the AI.

#### 5.2.3. Decommitment

At time T+1, employing the decryption key TSK posted by AI, both the platform and all users, can recover their encrypted sensing profile $e_{i}$ as well as their random string $r_{i}$. Applying the platform decryption key, the platform also recovers random values $r_{1},\cdots ,r_{k}$ for the verification of correctness and the pair for computing the auction’s results. The platform then computes the set of winners and their corresponding payments from the platform based on the above auction mechanism under the given budget. The platform posts the winner’s identity and the encrypted payment information so that any party can verify the correct results on the bulletin board.
**Algorithm 4** PVI-H // Privacy-Protection Verifiable Incentive Mechanism for Crowdsensing Applications with Homogeneous Sensing Jobs or Heterogeneous Sensing Jobs**Input:** User set U, the budget constraint *B*.**Output:**  *S*.  // Phase 1: Winner selection1:Initialize: Each user *i* receives his encrypted sensing profile (${\tilde{b}}_{i}$, ${\tilde{t}}_{i}$) by using the OT technology in [17], and submits their commitments to the platform; At time T+1, the platform makes a decommitment and sorts users in U i.e., ${\tilde{b}}_{1}<{\tilde{b}}_{2}\cdots <{\tilde{b}}_{\left|U\right|}$; S←∅; i=1;2:$b_{1}\leftarrow OPENS^{-1}\left({\tilde{b}}_{1}\right)$;3:**while** $b_{i}\leq B/\sum_{j\in S}f_{j}$ **do**4: $f_{i}\leftarrow 1$;5: **if** jobs are heterogeneous **then**6:  $f_{i}\leftarrow \min\left\{OPENS^{-1}\left({\tilde{l}}_{i}\right),\tau_{i}\right\}$, where $\tau_{i}=\left\lfloor \left(B-b_{i}\sum_{j\in S}f_{j}\right)/b_{i}\right\rfloor$;7: **end if**8: S←S∪i;9: i←i+1;10: $b_{i}\leftarrow OPENS^{-1}\left({\tilde{b}}_{i}\right)$;11:**end while**// Phase 2: Payment determination12:**for** each user i∈S **do**13: $l_{i+1}\leftarrow OPENS^{-1}\left({\tilde{l}}_{i+1}\right)$;14: **if** j≤i−1 **then**15:  Pay $p_{j}f_{j}$ to user *j*;16: **end if**17: **if** j=i **then**18:  $p_{j}\leftarrow \min\left\{B/\sum_{j\in S}f_{j},b_{i+1}/l_{i+1}\right\}$; Pay $p_{j}f_{j}$ to user *j*;19: **end if**20:**end for**21:**return** *S*


(a) **Winners Selection:** In this stage, the winners’ selection’s goal is to find the biggest integer *k* so that $b_{k}\leq B/\sum_{i=1}^{k}f_{i}$ holds, thereby obtaining the set of winners. Firstly, the platform first recovers the bids ${\tilde{b}}_{i}$ on the bulletin board and then sorts encrypted bids from all users and resorts to the AI to fetch the original value $b_{1}$ of ${\tilde{b}}_{1}$: $b_{1}=OPENS^{-1}\left({\tilde{b}}_{1}\right)$. If $b_{i}\leq B/\sum_{j\leq i}f_{j}$ holds, then users with the rank 1,2,⋯,i are winners, thus, for the platform, privacy leakage does not exist. Otherwise, the largest integer k=i−1. When user *i* is added to the winners’ set, the platform calculates his assignments ${\tilde{f}}_{i}\leftarrow \min\left\{OPENS^{-1}\left({\tilde{l}}_{i}\right),{\tilde{\tau }}_{i}\right\}$. The process is repeated until the above goal is achieved. The set of winners {1,2,⋯,k} is found. Notable, when we determine the largest *k*, if $b_{i}\leq B/\sum_{j\leq i}f_{j}$ does not hold, the k+1-th user’s bids and assignments, i.e., its sensing profile, may be disclosed (see Figure 2, step ④). Since in our crowdsensing applications, we assume that the number of users is much larger than the number *k*. As such, our scheme satisfies *k*-anonymity. So, neither the AI, nor the platform, can identify any user’s sensing profile with the probability higher than 1/k. The detailed description is illustrated in Algorithm 4.

(b) **Payment Decision:** In the payment determination phase, the platform pays $p_{j}f_{j}$ to user *j* for j≤i. Similarly, for each winner i∈S, the payment of per sensing job, i.e., $p_{i}$, is given in Algorithm 4. In particular, our payment scheme is applicable to homogeneous and heterogeneous sensing job models (see Figure 2, step ⑤).

#### 5.2.4. Verification

**Figure 3:**
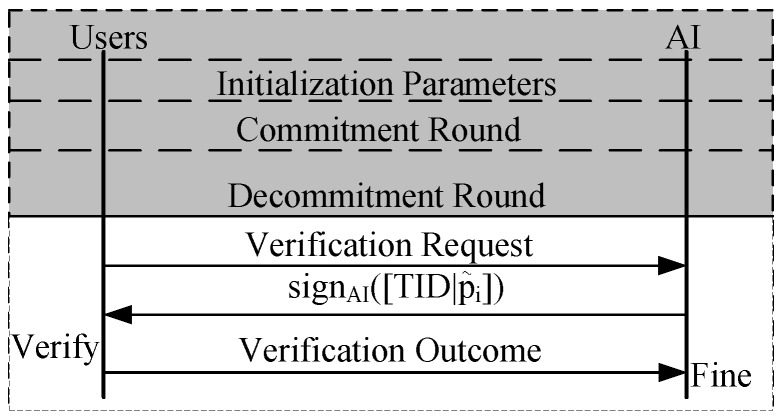
Our verifiable phase for homogeneous and heterogeneous jobs.

Since the above incentive mechanism guarantees the truthfulness for users, we only need to verify the payment correctness of the platform, that is, any of the users can verify the outcome of the auction on his own. The detailed descriptions are given as follows. Firstly, user *i* requests AI to verify the payment outcome with the probability α. After the AI receives the request, he asks for the random value $r_{i}$ of each user’s $e_{i}$. Then he derives each user $e_{i}$’s ${\tilde{b}}_{i},{\tilde{l}}_{i}$ by decrypting $e_{i}$ on the bulletin board with $r_{i}$, thereby obtaining the payment according to the above auction details and the information from the bulletin board. He sends the encrypted payment $f_{i}p_{i}$ and his assignments $f_{i}^{\prime }$ to the user *i* to verify the correctness of the outcomes from the platform,thereby obtaining the user’s feedback to determine whether to fine the platform (see Figure 3). Analysis in the following section shows that the platform operates correctly and does not try to cheat.

### 5.3. Design Privacy-Preserving Details for Submodular Sensing Jobs

Different from the above mechanism, for the submodular sensing job model, we need to overcome the challenge of protecting platform’s privacy, i.e., the privacy of the current winners’ set, by using the above-mentioned MPEP method and homomorphic encryption scheme. The detailed descriptions are described below.

#### 5.3.1. Initialization

The platform sends the following information to the AI: the deadline *T*, and its task identifier TID, the timed-lapse encryption key TPK applied by all users in commitments, and the description of the mechanism. If the AI accepts them, he will set the probability of the auditions from the users as α so that $\alpha \geq p_{max}/\left(F+p_{max}\right)$, where $p_{max}$ and *F* are the maximal payment and fine paid from the platform respectively, and sends signed α and signed auction details to the platform. If it is accepted by the platform, it will be posted them to the bulletin board. A set of possible marginal utilities per bid is defined as $\beta =\left\{\beta_{1},\beta_{2},\cdots ,\beta_{z}\right\}$, where $\beta_{1}<\beta_{2}<\cdots <\beta_{z}$ holds, and requires that each user *i*’s marginal utility per bid $\omega_{i}\in \beta$. The AI maps each $\beta_{i}$ to $\gamma_{i}$, while the rank is kept, i.e., $\gamma_{1}<\gamma_{2}<\cdots <\gamma_{n}$ holds. Similarly, each user’s marginal utility per bid is transformed by applying the order preserving encryption scheme (OPES) [18] for preserving their ranks. Assume that the above-mentioned AI can bootstrap the crowdsensing application and and all of the previous data signatured by the platform $sign_{p}$ can be posted on the bulletin board. Then it constructs three dynamic lists for the verification of payments’ correctness initiated by each user. The first list $l_{i}^{w}$ for user *i* is used to put his marginal utility per bid $\omega_{i}\left(S\right)$ for the winner determination phase. The second list $l_{i}^{p}$ is used to put his marginal utility per bid $\omega_{i}\left(T\right)$ for the payment determination phase. The last dynamic list $l_{i}^{S}$ is constructed for each winner.

#### 5.3.2. Commitment Round for Winner and Payment Determination

**Figure 4:**
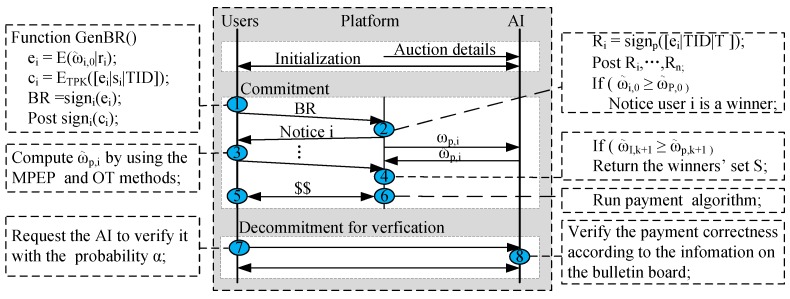
Our privacy-preserving verifiable framework for submodular jobs.

User *i* initially selects a subset $\Gamma_{i}$ of assignments and a bid $b_{i}$ according to his valuation he preferences. Each user *i* initially computes his marginal utility $U_{i}\left(\emptyset \right)$, thereby obtaining his marginal utility per bid $\omega_{i}\left(\emptyset \right)$. Then he interacts with the AI by using the OT technology in [17], thereby receiving ${\tilde{\omega }}_{i,0}\left(\emptyset \right)$, where the subscript 0 denotes the cardinality of the current winners’ set is equal to 0, and ${\tilde{\omega }}_{i,0}\left(\emptyset \right)$ is the rank-preserving-encrypted value of $\beta_{x}$. Then each user *i* encrypts it as $e_{i}=E\left({\tilde{\omega }}_{i,0}|r_{i}\right)$ by applying the platform’s Paillier encryption key $K_{pub}$ and a randomly value $r_{i}$. Then user *i* commits $c_{i}=E_{TPK}([e_{i}|s_{i}\left|TID]\right)$, where $s_{i}$ is a bit string randomly generated for the proof of correctness. Finally, the user *i* signs the commitment $c_{i}$ and the encrypted value $e_{i}$. Then he adds $sign_{i}\left(c_{i}\right)$ to the list $l_{i}^{w}$ on the bulletin board and sends $sign_{i}\left(e_{i}\right)$ to platform. Receiving all users’ values $sign_{i}\left(e_{i}\right)$, the platform decrypts and sorts them, thereby obtaining the user *i* with the maximal encrypted marginal utility per bid. Moreover, the platform enters the following winner determination phase. The more detailed illustration is given in Figure 4.

(a) **Winner Determination:** Firstly, the platform applies the homomorphic encryption scheme to compute the utility U(S∪{i}) according to Algorithm 3, thereby obtaining $\omega_{p,0}=U\left(S\cup \left\{i\right\}\right)/B$. By using the OT technology in [17], the platform interacts with the AI, and receives the encrypted ${\tilde{\omega }}_{p,0}$. The platform makes a commit $c_{p,0}=E_{TPK}([{\tilde{\omega }}_{p,0}|s_{p}\left|TID]\right)$, where $s_{p}$ is a bit string randomly generated for the proof of correctness. Signing it, $sign_{p}\left(c_{p,0}\right)$, the platform adds it to the list $l_{p}^{w}$ on the bulletin board. If ${\tilde{\omega }}_{i,0}\left(\emptyset \right)\geq {\tilde{\omega }}_{p,0}$, the platform will give user *i* a notice that he is a winner. Then the user returns an acknowledgement and his encrypted $\Gamma_{i}$ and $b_{i}$ by using the platform’s public key. Receiving the acknowledgement, the platform adds user *i* to winners’ set *S* (see the line 5 of Algorithm 5.) and notifies each user j∈U\S to compute his encrypted marginal utility per bid, i.e., ${\tilde{\omega }}_{j,1}$, by using the same method as the computation of ${\tilde{\omega }}_{i,0}$. These users also add their signed commitments to their corresponding lists $l_{j}^{w}$ on the above-defined bulletin board. When the platform receives all these ${\tilde{\omega }}_{j,1}$, it sorts them, thereby knowing which user has the maximal encrypted marginal utility per bid. The process is repeated until the (k+1)-th user’s ${\tilde{\omega }}_{i,k+1}\left(\emptyset \right)<{\tilde{\omega }}_{p,k+1}$. Finally, we obtain the winners’ set that consists of *k* users.
**Algorithm 5** Winner Determination for Sensing Submodular Jobs**Input:** User set U, the budget constraint *B*.**Output:** The winners’ set *S*.1:S←∅; For every j∈U, the platform recovers ${\tilde{\omega }}_{j,0}\left(S\right)$ by using the decryption algorithm, and sorts all these values in a decreasing order, thereby obtaining the user *i* with the maximal encrypted marginal utility per bid, i.e., $i\leftarrow \arg\max_{j\in U}{\tilde{\omega }}_{j,0}\left(S\right)$;2:The platform obtains ${\tilde{\omega }}_{p,0}\left(S\right)$ by using the OT technology in [17] and Algorithm 3, and adds a signed commitment to the list $l_{p}^{w}$ on the bulletin board;3:**while** ${\tilde{\omega }}_{i,0}\left(\emptyset \right)\geq {\tilde{\omega }}_{p,0}$ **do**4: The platform notices that user *i* is a winner;5: Receiving an acknowledgement, the platform adds user *i* to the winner set *S*, i.e., S←S∪{i};6: Notify each user j∈U\S to compute his encrypted marginal utility per bid, i.e., ${\tilde{\omega }}_{j,|S|}$, by using the same method as the computation of ${\tilde{\omega }}_{i,0}$; Obtaining all these encrypted marginal utilities per bid, the platform finds the user *i* so that $i\leftarrow \arg\max_{j\in U\backslash S}\left({\tilde{\omega }}_{j,|S|}\left(S\right)\right)$;7: The platform obtains ${\tilde{\omega }}_{p,|S|}\left(S\right)$ by using the OT technology in [17] and Algorithm 3, and adds a signed commitment to the list $l_{p}^{w}$ on the bulletin board;8:**end while**9:**return** *S*

(b) **Payment Determination:** At this stage, the encrypted values from the above OT algorithm cannot support the preserving rank under the multiplication operation. To address this challenge, we introduce the homomorphic encryption schemes, which enable multiplication operation of encrypted values without disclosing privacy about the values and the computation’s result. Firstly, at time *T*, for each winner i∈S, its payment computation from the platform is given in the following description. The platform initializes the user set $U^{\prime }$ and set T by using $U^{\prime }\leftarrow U\backslash \left\{i\right\}$ and T←∅. Differentiating from the above winner set, we refer to T as a referenced winner set. Each user $j\in U^{\prime }\backslash T$ initially computes his marginal utility $U_{j}\left(\emptyset \right)$, thereby obtaining his encrypted marginal utility per bid $e_{j,0}=E_{AI}\left(\omega_{j,0}\left(\emptyset \right)\right)$ by using the AI’s homomorphic encryption public key. He makes a commit $c_{j,0}$ by using the above method. Finally, user *j* signs this commitment $c_{j,0}$ and the encrypted value $e_{j,0}$. Then he adds $sign_{j}\left(c_{j,0}\right)$ to the list $l_{j,i}^{p}$ on these bulletin board (meaning that the list is used to put user *j*’s commitment for the computation of user *i*’s payment) and sends the $sign_{j}\left(e_{j,0}\right)$ to the platform. Receiving the values of all users in $U^{{}^{\prime }}$, the platform sorts them, thereby obtaining the user $i_{j}$ with the maximal encrypted marginal utility per bid (i.e., $e_{i_{j},0}$). Then the platform notices that user $i_{j}$ is a referenced winner and requests user *i* for obtaining the $E_{AI}\left(U_{i(j)}\right)$. After user $i_{j}$ receives the request, he computes the value $E_{AI}\left(U_{i(j)}\right)$ by applying the above MPEP and AI’s encryption public key. Signing it, he sends the signed $E_{AI}\left(U_{i(j)}\right)$ to the platform. According to the homomorphic encryption, we have $E_{AI}\left(b_{i(j)}\right)=E_{AI}\left(U_{i(j)}\cdot b_{i_{j}}/U_{i_{j}}\right)=E_{AI}\left(U_{i(j)}\cdot 1/\omega_{i_{j}}\right)=E_{AI}{\left(U_{i(j)}\right)}^{1/e_{i_{j}}}$. Similarly, we can obtain $E_{AI}\left(\eta_{i(j)}\right)=E_{AI}\left(U_{i(j)}\cdot B/U\left(T_{j-1}\cup \left\{i\right\}\right)\right)=E_{AI}\left(U_{i(j)}\cdot 1/\omega_{p,j}\right)=E_{AI}{\left(U_{i(j)}\right)}^{1/e_{p,j}}$, where $e_{p,j}$ means the encrypted marginal utility per bid when there are *j* referenced winners. Since user *i* is a true winner, the platform knows his bid and sensing preference $\Gamma_{i}$. Thus, the platform can compute the value $e_{p,j}$. Receiving the value $E_{AI}\left(U_{i(j)}\right)$, the platform can obtain $E_{AI}\left(b_{i(j)}\right)$ and $E_{AI}\left(\eta_{i(j)}\right)$. Furthermore, the interim payment can be obtained by using the homomorphic encryption comparison operation. Subsequently, the platform adds user $i_{j}$ to the referenced winners’ set. The process is repeated until the $\left(k^{\prime }+1\right)$-th user’s $e_{i_{j},k^{{}^{\prime }}+1}\left(T_{k^{{}^{\prime }}}\right)<e_{p,k^{\prime }+1}\left(T\right)$. Finally, we obtain the payment of winner *i*. Other winners’ payments are computed by adopting the same method as the winner *i*’s payment. The details are given in Algorithm 6.
**Algorithm 6** Payment Determination for Sensing Submodular Jobs**Input:** User set U, the budget constraint *B*, the set of winners *S*.**Output:** (U, *p*).1:**for** each user i∈U **do**2: ${\hat{p}}_{i}\leftarrow E_{AI}\left(0\right)$;3:**end for**4:**for all** user i∈S **do**5: $U^{\prime }\leftarrow U\backslash \left\{i\right\}$; the referenced winners’ set T←∅;6: **repeat**7:  Every $j\in U^{{}^{\prime }}$ computes his encrypted marginal utility per bid $e_{j,|T|}=E_{AI}\left(\omega_{j,|T|}\left(T\right)\right)$ by using AI’s homomorphic encryption public key for sending to the platform, and adds a signed commitment $sign_{j}\left(c_{j,|T|}\right)$ to the list $l_{j,i}^{p}$ on these bulletin board; Receiving these encrypted values, the platform sorts them in a decreasing order, thereby obtaining the user $i_{j}$ with the maximal encrypted marginal utility per bid, i.e., $i_{j}\leftarrow \arg\max_{j\in U^{\prime }\backslash T}e_{j,|T|}\left(T\right)$;8:  Notice that user $i_{j}$ is a referenced winner and requests user *i* for obtaining the $E_{AI}\left(U_{i(j)}\right)$;9:  According to the description of Section 5.3.2, the platform computes $E_{AI}\left(b_{i_{j}}\right)$ and $E_{AI}\left(\eta_{i(j)}\right)$ by applying the Paillier cryptosystem and its homomorphic property in [46]; Obtain ${\hat{p}}_{i}\leftarrow \max\{{\hat{p}}_{i},\min\left\{E_{AI}\left(b_{i_{j}}\right),E_{AI}\left(\eta_{i(j)}\right)\right\}$;10:  $T_{j-1}\leftarrow T$; $T\leftarrow T\cup \{i_{j}\}$;11: **until** $e_{i_{j},k^{\prime }+1}\left(T_{k^{\prime }}\right)<e_{p,k^{\prime }+1}\left(T\right)$ or $T=U^{{}^{\prime }}$12: The platform requests the AI for obtaining the payment, i.e., $p_{i}=D_{AI}\left({\hat{p}}_{i}\right)$, where $D_{AI}$ denotes the decryption by using the AI’s private key;13:**end for**14:**return** (U, *p*)

#### 5.3.3. Decommitment Round for Verification

Since the mechanism itself is truthful, i.e., each user always submits his true cost, we only demonstrate that any user can check the correctness of the platform’s payment on his own.

**Verification:** The verification process is similar to the above description (see Figure 3). The only difference is that three dynamic lists in the bulletin board are used to recover associated values for the payment computation of each user. Generally speaking, some user initially sends the request of verification to the AI with the probability α. Receiving the request, the AI runs the algorithm description on the bulletin board by inputting the values in the three lists until the payment is obtained. For more details of verification, we refer readers to Section 5.2.4 and Figure 3.

#### 5.3.4. Verifiable Privacy-protection Incentive Mechanism for Sensing Submodular Jobs

In our truthful verifiable privacy-protection incentive mechanism for sensing Submodular jobs, the platform will output a winner *i*’s payment. Firstly, some initial parameters are specified by the platform. Then, the platform performs the winner’s selection and the payment determination in turn. Once the payment is finished by the platform, user *i* will request AI to verify the payment correctness of platform with the probability α. The details are illustrated in Algorithms 5–7.
**Algorithm 7** PVI-S // Privacy-Protection Verifiable Incentive Mechanism for Crowdsensing Application with Sensing Submodular Jobs**Input:** User set U, the budget constraint *B*.**Output:**  (U, *p*).1:Initialize the auction information and encryption tools;2:Choose the winners by applying the algorithm;3:Finish the payment for each winner;4:The user requests the AI to verify the payments with the probability α;5:**return** (U, *p*)

## 6. Privacy, Verifiability and Revenue Analysis

### 6.1. Privacy of Users and Platform

Our mechanisms’ private information include users’ privacy and platform’s privacy, i.e., the sensing profile privacy of users and the current winners’ set privacy of the platform. Assume that there are two kinds of adversaries: adversarial users and adversarial platform or AI. The specific analysis is given as follows.

**Lemma** **1.** The mechanisms PVI-H and PVI-S are privacy-preserving for users.

**Proof.**  We only need to consider two cases in which the privacy of each user *i* may be leaked as follows. The first case is for the adversarial platform or AI. In the two mechanisms, the platform performs the winners’ selection, and only can know the (k+1)-th user’s sensing profile $P_{k+1}$, but does not know which user it belongs to. In the stage of verification, similarly, the AI also knows the (k+1)-th user’s sensing profile $P_{k+1}$, and does not know which user it belongs to. The AI and platform only know the encrypted sensing profile, but have no way to decrypt any of them. No other party can obtain even more information than the platform or AI. On the one hand, user *i* gets his sensing profile $P_{k+1}$ through a 1-out-of-*z* OT from the AI, who is unknown of which sensing profile have been accessed by the user. User *i* sends the encrypted sensing profile to the AI, who cannot decrypt the encrypted sensing profile without knowing the private key for the asymmetric encryption scheme. Even if the AI may know the (k+1)-th user’s sensing profile later when the platform consults him, he still cannot infer his user owing to the random number. Thus, the AI cannot know the user of (k+1)-th user. Additionally, although the platform can obtain the (k+1)-th user’s sensing profile later, he can only reversely map the encrypted (k+1)-th user’s sensing profile to the original (k+1)-th user’s sensing profile with the help of the AI. However, the platform still cannot derive the user, to which (k+1)-th user’s sensing profile belongs out of at least *k* members according to the Theorem 3.2 in [4] due to a large number of users much larger than *k* existing in the crowdsensing applications. Therefore, neither the AI, nor platform, can know any user’s sensing profile with the probability higher than 1/k, thereby guaranteeing *k*-anonymity.

The second case is for an adversarial user. In the two mechanisms, an adversarial user *j* does not learn side information during our mechanisms no matter he is a winner or not. All he learns from the two mechanisms are included in the valid auction’s Output, i.e., for an adversarial user *j*’s advantage $adv_{P_{i}}$ are all equal to 0 for all i≠j.

Putting them together, the lemma holds. ☐

Besides, in the following lemma, the privacy preservation performance of the platform will be analyzed in details.

**Lemma** **2.** For the current winners’ set S and referenced winners’ set T of the platform (the privacy of the platform), an adversarial user j’s advantage, i.e., $adv_{S}$ and $adv_{T}$, are equal to 0. In other words, the mechanisms PVI-H and PVI-S are privacy-preserving.

**Proof.**  For the current winners’ set *S* and referenced winners’ set T, only platform and AI learn the two sets and each user learns nothing. Since the AI is semi-honest, and only check the platform randomly, adversarial users gain no useful information on the two sets from the communication strings. Thus, the priori probability is same as the posterior probability, i.e., $Adv_{S}=P_{r}\left[S|C,Output\right]-P_{r}\left[S|Output\right]=0$ and $Adv_{T}=P_{r}\left[T|C,Output\right]-P_{r}\left[T|Output\right]=0$. Thus, the mechanisms PVI-H and PVI-S are privacy-preserving for the platform. Thus, the lemma holds. ☐

Putting these lemmas together, the following theorem will be derived.

**Theorem** **1.** The mechanisms PVI-H and PVI-S are privacy-preserving.

### 6.2. Verifiable Correctness of Payments

**Lemma** **3.** The users in the mechanisms PVI-H and PVI-S is truthful.

**Proof.**  For the mechanism PVI-H, we can easily extend the outcome of the homogenous jobs presented by Singer et al. [4] the proof outcome to the heterogeneous jobs. For the mechanism PVI-S according to [6], since Algorithm PVI-S is designed based on the MSensing mechanism of [6], they have demonstrated the truthfulness of the mechanism, our mechanism PVI-S is also truthful for users in crowdsensing applications. Thus, the lemma holds. ☐

Generally speaking, the verifiability issue includes the Verifiability of users’ sensing profile and platform’s payment. From the above lemma 3, we know that users’ bid is truthful. Besides, each user’s subset of assignments is fixed in our mechanisms. Thus, each user’s sensing profile is truthful. Therefore, we only need to guarantee the verifiable correctness of payments from the platform. Furthermore, we have the following lemma.

**Lemma** **4.** The two proposed mechanisms, i.e., PVI-H and PVI-S, are correct for a rational platform.

**Proof.**  Correctness of both PVI-H and PVI-S, follows the assumption that the platform is rational and the paid fine is high enough when checked cheating. If his expected utility when complying with both PVI-H and PVI-S is higher than the one from his deviation he will abide by the algorithm, as such the proposed algorithms i.e., PVI-H and PVI-S, will be correct. We will show the probability α that the platform’s incorrect payment will not be checked by the user with the help of the AI, set by the two algorithms i.e., PVI-H and PVI-S, ensures that the platform’s expected utility is non-positive. The detailed derivation is given as follows. $\alpha \geq p_{max}/\left(f+p_{max}\right)\Rightarrow \left(1-\alpha \right)p_{max}-\alpha f\leq 0$. Considering the platform’s expected utility, i.e., $\left(1-\alpha \right)V_{+}+\alpha V_{-}$, where $V_{+}$ denotes the platform’s utility when it gives incorrect payment but is not checked by the users, and $V_{-}$ denotes the platform’s utility when it gives incorrect payment but is checked by the users [12]. Again, $p_{max}\geq V_{+}$ and $-f=V_{-}$, according to the outcome of the above derivation, further, we have $\left(1-\alpha \right)V_{+}+\alpha V_{-}\leq \left(1-\alpha \right)p_{max}-\alpha f\leq 0$. Thus, if the platform does not comply with the algorithm PVI-H, its expected utility is non-positive. As such, for a rational platform, it is willing to abide by the rules of both PVI-H and PVI-S, and gives a correct payment for every user. Finally, the lemma holds. ☐

Putting these lemmas together, we have the following theorem.

**Theorem** **2.** The mechanisms PVI-H and PVI-S are verifiable correctness of payments.

### 6.3. Revenue of Platform

**Lemma** **5.** The mechanisms in Section 4 are O(1)-competitive in maximizing the revenue of the platform.

**Proof.**  To quantify the revenue of the platform running the mechanisms in Section 4, we compare their revenue with the optimal revenue: the obtainable revenue for the offline scenario where the platform has full knowledge of users’ sensing profiles. A mechanism is O(1)-competitive if the ratio of the mechanism’s revenue to the optimal revenue is a constant factor approximation. According to the Theorem 3.4 in [4] and Theorem 4.5 in [4], we know that the mechanisms in Section 4 are budget feasible constant-approximation mechanisms, and no budget feasible mechanism could do better than mechanisms of Section 4 in maximizing the homogeneous, heterogeneous sensing revenue and submodular sensing revenue of the platform. Thus, the lemma holds. ☐

Furthermore, different from the mechanisms in Section 4, mechanisms PVI-H and PVI-S mainly apply the order preserving encryptions and the OT operations. However, these encryptions and operations in mechanisms PVI-H and PVI-S do not change the allocation and payment rules of the mechanisms in Section 4. Thus, mechanisms PVI-H and PVI-S keep the same revenue as the mechanisms in Section 4, thereby obtaining the following theorem.

**Theorem** **3.** The mechanisms PVI-H and PVI-S have the same revenue as the generic one without privacy protection.

## 7. Performance Evaluation

In this section, the communication and computation overhead are analyzed to show our mechanisms are both scalable and efficient. Most of the complexities are linear to the users’ number or the assignments’ number, which allows huge number of users or the number of assignments. Meanwhile, extra data transmission and the run time introduced by our mechanisms are almost negligible.

### 7.1. Simulation Setup

The two PVI mechanisms were run on a PC with 1.7 GHz CPU and 8 GB memory. Each measurement is averaged over 100 instances. We set the order *p* of the integer group $Z_{p}$ as a 1024-bit prime number, thereby users can get 128 bits of rank-keeping encrypted value according to the OT technology.

### 7.2. Performance Evaluation for the PVI-H Mechanism

#### 7.2.1. Bulletin Board Storage Complexity

We require the bulletin board to store the auction details and dynamic lists used to store the parameters or values accessed by the platform and AI. In each list, there are only few elements. Thus, the storage’s complexity is θ(n), in which *n* is the users’ number.

#### 7.2.2. Communication Overhead

The communication overhead based on the data transmission is illustrated in Table 2, in which $l_{bit}$ is the *p*’s bit length (i.e., the order of the integer group $Z_{p}$). A detailed explanation of the order of the integer group $Z_{p}$ can be found in random numbers in sorting of [10].

Note that the computation of accumulated assignments in the winner determination phase is executed until the platform finds the largest *k* so that $b_{i}\leq B/\sum_{j\leq i}t_{j}$ holds. Based on random numbers’ sorting of [10], finding the order of the integer group $Z_{p}$ of bit length $l_{bit}$ needs $O(l_{bit})$. Thus, the average communication rounds for the platform should be much less than O(mn) (*m* is the number of different assignments), which means that the real communication overhead will be much less than the worst case $O(mnl_{bit})$. In this stage, each user only sends the sensing data to the platform and AI, therefore the sending overhead of each user is $O(l_{bit})$. However, the receiving overhead of each user is $O(nl_{bit})$. It is because each user needs to receive the information from all user.

For the overhead in the payment determination, according to Algorithm 4, the sending overhead of the platform takes $O(nm^{2}l_{bit})$ time. However, the receiving overhead of the platform takes $O(ml_{bit})$ time. It is because it only need receiving at most m assignments from winners.

For the overhead of verification, both the sending and receiving overheads are $O(l_{bit})$. It is because the verification is executed only from some user. According to Section 5.2.4, the verification sending and receiving overheads of both the platform and AI are $O(nl_{bit})$. It is because they send and receive *n* commitments.

**Figure 5:**
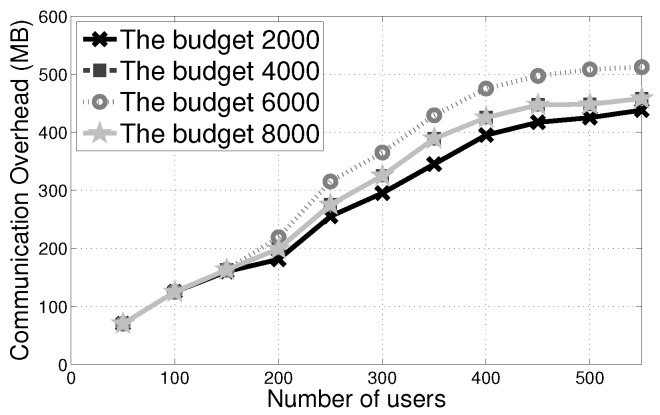
Communication overhead of PVI-H with different budgets.

Since the verification from the AI does not need the communication for the computation of accumulated assignments, it only requires information from the existing bulletin board. Thus their communication overhead is negligible. Figure 5 indicates that the overall communication overhead induced by Algorithm PVI-H. Obviously, the communication overhead is mainly from the OT.

#### 7.2.3. Computation Overhead

Since for the winner selection, the payment determination and verification, each user only needs to do the computation operations of constant times. Thus, the computation overhead of users is O(1). For the platform, according to Algorithm 4, in the winner selection stage, the while-loop (lines 3–11) of Algorithm 4 takes $O(nm^{2})$. In the payment determination stage, the for-loop (lines 12–20) of Algorithm 4 takes $O(nm^{3})$. In the verification stage, since the platform need not to make the computation operation. Hence, we can derive the computation overhead given in Table 3. Finally, the AI only needs to do small works for the winner selection and payment determination, therefore the overhead in the two stages is O(1). The overhead’s analysis of the verification stage of the AI is similar to the one of the payment determination stage of the platform, so its overhead is $O(nm^{3})$.

Put these together, the detailed overhead of the computation is illustrated in Table 3.

The PVI-H mechanism consists of the winner selection phase and the payment determination phase. The winner selection phase mainly includes the OT, the sorting, the blind signature generation and the computation of accumulated assignments; the payment determination phase mainly includes the payment calculation of the platform. Compared with the above parts, since verifying the payment are run on the bulletin board, we can neglect the computation overhead of the verification. Next, we will in turn estimate their run time.

In the PVI-H mechanism, the signer’s run time is 19 ms, while the runtime of one pair of the Nyberg-Rueppel blindly signature is 11 μs(microseconds) on average.

The computation overhead of the sorting and OT, and the effects of different budget constraints for each winner are similar to our previous work in [17]. Thus, under the same conditions with [17], a single computation requires 0.4μs on average in the same conditions as [17], thereby the computation overhead is very small. Similarly, the run time of the calculation of accumulated assignments and payment for various number of assignments and payment is almost negligible.

### 7.3. Performance Evaluation for the PVI-S Mechanism

#### 7.3.1. Bulletin Board Storage Complexity

We require the bulletin board to store the auction details and three dynamic lists of each user used to store the values accessed by the AI. In each list, there are only few elements. Thus, the storage’s complexity is θ(n).

#### 7.3.2. Communication Overhead

The communication overhead in terms of transmitted bits is illustrated in Table 4. The communication overhead analysis is similar to Section 7.2.2. The only difference is the difference between Algorithms 4 and 6. Note that the marginal-utility-per-bid computation in the winner selection and payment determination is executed until the platform and AI finish the winner selection and the payment determination. Because there are *m* different assignments, and each winner should at least contribute one new assignment to be chosen, the winners’ number in the payment determination phase is at most *m*. Thus, the average communication rounds for the platform should be much less than $O(m^{2})$, which means the practical communication overhead will be much less than the worst case $O(m^{2})$. Besides, the MPEP’s introduction for the marginal-utility-per-bid computation, makes the communication overhead of each user different with the PVI-H mechanism (see Table 4).

#### 7.3.3. Computation Overhead

The computation overhead analysis is similar to Section 7.2.3. Hence, the overhead of the computation is illustrated in Table 5.

In general, the PVI-S mechanism consists of the winner selection phase, the payment determination phase, and the verification phase. The winner selection phase includes users’ blind signature, the sorting of the platform and the computation of marginal utility per bid. The payment determination phase includes the sorting of the platform and the computation of marginal utility per bid. In the verification phase, compared with the above parts, since verifying the payment are run on the bulletin board, we can neglect the computation overhead of the verification. Now, their run times are analyzed respectively.

**Figure 6:**
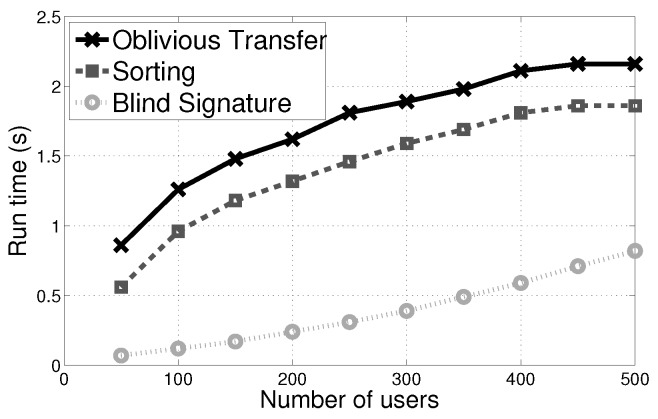
Run time of the sorting, the OT and the blind Signature of PVI-S with the users’ number when the budget’s value is 2000.

**Figure 7:**
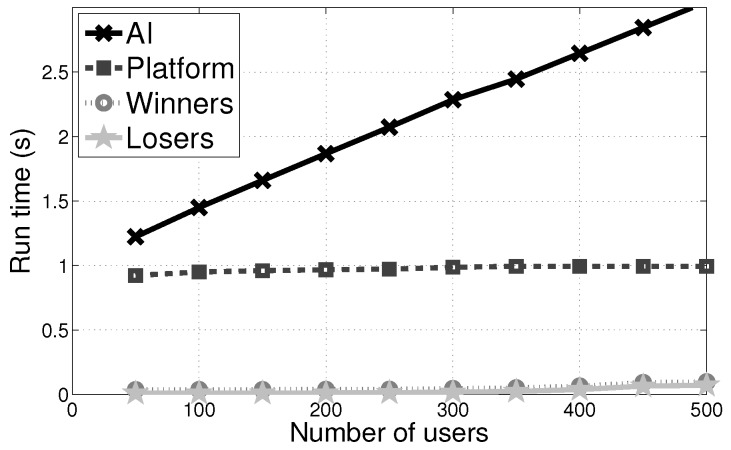
Run time of AI, the platform, losers and winners of PVI-S with the users’ number when the budget’s value is 2000.

**Figure 8:**
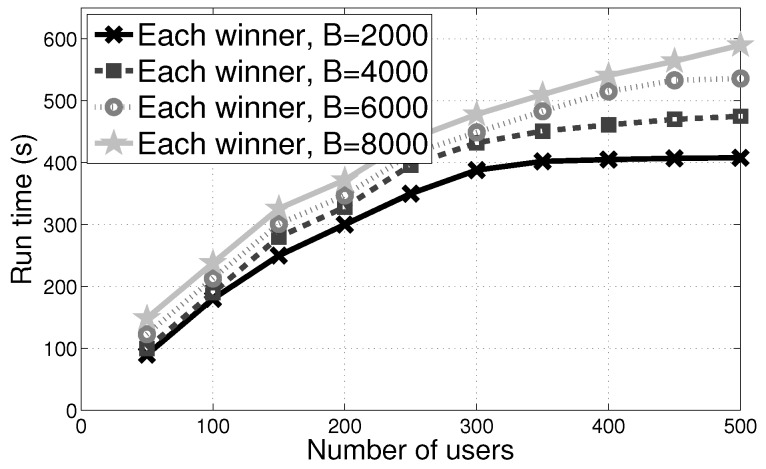
Limited budget effect of PVI-S on the computation overhead.

(a) **Sorting, OT and Blind Signature:** The PVI-S mechanism’s run time of a pair of the Nyberg-Rueppel signature including the AI, platform and users is 28 milliseconds on average. Further, we also evaluated the run time of the OT and the final sorting based on the encrypted values. We observed that the computation overhead of the signature is negligible when compared with the one of the OT and sorting. Users in the PVI-S have much less run time since they only generate the communication strings (ciphertexts) (see Figure 6). 

(b) **Computation of AI, Platform, Winners and Losers:** We compared the computation overhead of the AI, the platform, winners and losers in Figure 7 when the budget value is 2000. We observed that the computation overhead increases with the budget constraint and at last they were kept in a stable constant value respectively. It is because that at this moment the PVI-S mechanism reached saturation point.

(c) **Limited Budget Effect on Computation Overhead:** To assess the effect of different limited budget on computation overhead of winner *i*, we calculated the average computation overhead of each winner under different budget values respectively. We noted that the overall computation overhead had an increment with the winners’ number reached a stable value at last (see Figure 8). Computation overhead is very small, therefore each user’s overhead induced by the PVI-S mechanism also can be used to mobile devices.

## 8. Concluding Remarks

In this paper, we design two privacy-preserving verifiable incentive mechanisms for crowdsensing applications. We not only handle users’ privacy protection and the platform by using the OPES and OT, but also construct a verification scheme to ensure the payment correctness of the platform by using the signature technology and the bulletin board. We preserve the rank of the encrypted values by using the OPES scheme. Furthermore, we prevent bid repudiation by employing a TLC service. No party, including the platform, receives any information about bids before the mechanism closes, and no user is able to change or repudiate any sensing profile. Finally, we design and analyze the two mechanisms. Results from theory analysis and experiments show that our verifiable privacy-protection incentive mechanisms have the same results as the generic one without privacy protection and also apply for mobile devices in crowdsensing applications. As such, they can be extended to other truthful incentive mechanisms for real crowdsensing environments.

In the future, we will investigate the design of truthful incentive mechanisms when the huge amount of stream data appear in crowdsensing applications. Based on the results of this paper and these truthful incentive mechanisms oriented to the huge amount of stream data, we will furthermore explore the privacy-protection and verification issues of crowdsensing in the scenarios of the huge amount of stream data. 

## Figures and Tables

**Table 1:** Comparison of our work with competitive works.

Mechanism	Online or Offline	Homogenity	Heterogenity	Submodularity	Truthfulness	Verifiability	Private Protection
H-PVA [17]	Online	No	Yes	No	Yes	Yes	Yes
PVI-H	Offline	Yes	Yes	No	Yes	Yes	Yes
PVI-S	Offline	No	No	Yes	Yes	Yes	Yes
MSensing [6]	Offline	No	No	Yes	Yes	No	No
OMZ [19]	Online	No	No	Yes	Yes	No	No
Task pricing [5]	Online	No	Yes	No	Yes	No	No

**Table 2:** Communication overhead of PVI-H.

Winner Selection				
	Send	Receive	$\omega_{i}$ Computation	User Sorting
Users	$O(l_{bit})$	$O(nl_{bit})$	$O(nl_{bit})$	0
Platform	$O(nml_{bit})$	$O(nl_{bit})$	0	$O(n^{2}l_{bit})$
AI	$O(nl_{bit})$	$O(nl_{bit})$	0	$O(n^{2}l_{bit})$
**Payment Determination**				
Each winner	0	$O(l_{bit})$	$O(nl_{bit})$	0
Platform	$O(nm^{2}l_{bit})$	$O(ml_{bit})$	0	$O(m^{2}l_{bit})$
AI	$O(ml_{bit})$	$O(ml_{bit})$	0	$O(m^{2}l_{bit})$
**Verification**				
Each winner	$O(l_{bit})$	$O(l_{bit})$	0	0
Platform	$O(nl_{bit})$	$O(nl_{bit})$	0	$O(n^{2}l_{bit})$
AI	$O(nl_{bit})$	$O(nl_{bit})$	0	$O(n^{2}l_{bit})$

**Table 3:** Computation overhead of PVI-H.

	Winner Selection	Payment Determination	Verification
Users	O(1)	O(1)	O(1)
Platform	$O(nm^{2})$	$O(nm^{3})$	0
AI	O(1)	O(1)	$O(nm^{3})$

**Table 4:** Communication overhead of PVI-S.

Winner Selection				
	Send	Receive	$\omega_{i}$ Computation	User Sorting
Users	$O(l_{bit}\ln n)$	$O(l_{bit}\ln n)$	$O(l_{bit}n)$	0
Platform	$O(nml_{bit})$	$O(nl_{bit})$	0	$O(n^{2}l_{bit})$
AI	$O(nl_{bit})$	$O(nl_{bit})$	0	$O(n^{2}l_{bit})$
**Payment Determination**				
Each winner	0	$O(l_{bit})$	$O(nl_{bit})$	0
Platform	$O(m^{2}l_{bit})$	$O(ml_{bit})$	0	$O(m^{2}l_{bit})$
AI	$O(ml_{bit})$	$O(ml_{bit})$	0	$O(m^{2}l_{bit})$
**Verification**				
Each winner	$O(l_{bit})$	$O(l_{bit})$	0	0
Platform	$O(nl_{bit})$	$O(nl_{bit})$	0	$O(n^{2}l_{bit})$
AI	$O(nl_{bit})$	$O(nl_{bit})$	0	$O(n^{2}l_{bit})$

**Table 5:** Computation overhead of PVI-S.

	Winner Selection	Payment Determination	Verification
Users	O(1)	O(1)	O(1)
Platform	$O(nm^{2})$	$O(nm^{3})$	0
AI	O(1)	O(1)	$O(nm^{3})$

## References

[1] Foremski P., Gorawski M., Grochla K., Polys K. (2015). Energy-efficient crowdsensing of human mobility and signal levels in cellular networks. Sensors.

[2] Thiagarajan A., Ravindranath L., LaCurts K., Madden S., Balakrishnan H., Toledo S., Eriksson J. VTrack: Accurate, energy-aware road traffic delay estimation using mobile phones. Proceedings of the 7th ACM Conference on Embedded Networked Sensor Systems.

[3] Maisonneuve N., Stevens M., Niessen M.E., Steels L. (2009). NoiseTube: Measuring and mapping noise pollution with mobile phones. Information Technologies in Environmental Engineering.

[4] Singer Y. Budget feasible mechanisms. Proceedings of the IEEE Foundations of Computer Science.

[5] Singer Y., Mittal M. Pricing Tasks in Online Labor Markets. Proceedings of the 11th AAAI Conference on Human Computation.

[6] Yang D., Xue G., Fang X., Tang J. Crowdsourcing to smartphones: Incentive mechanism design for mobile phone sensing. Proceedings of the 18th Annual International Conference on Mobile Computing and Networking.

[7] Parkes D.C., Rabin M.O., Shieber S.M., Thorpe C. (2008). Practical secrecy-preserving, verifiably correct and trustworthy auctions. Electron. Commer. Res. Appl..

[8] Dong W., Dave V., Qiu L., Zhang Y. Secure friend discovery in mobile social networks. Proceedings of the IEEE 51st Annual IEEE Symposium on Foundations of Computer Science.

[9] Huang Q., Tao Y., Wu F. Spring: A strategy-proof and privacy preserving spectrum auction mechanism. Proceedings of the IEEE INFOCOM.

[10] Jung T., Li X.Y., Zhang L., Huang H. (2013). Efficient, Verifiable and Privacy-Preserving Combinatorial Auction Design. arXiv.

[11] Angel S., Walfish M. Verifiable auctions for online ad exchanges. Proceedings of the ACM SIGCOMM.

[12] Catane B., Herzberg A. Secure Second Price Auctions with a Rational Auctioneer. Proceedings of the 2013 International Conference on Security and Cryptography (SECRYPT).

[13] Ganti R.K., Pham N., Tsai Y.E., Abdelzaher T.F. PoolView: Stream privacy for grassroots participatory sensing. Proceedings of the 6th ACM Conference on Embedded Network Sensor Systems.

[14] Shi J., Zhang Y., Liu Y. Prisense: Privacy-preserving data aggregation in people-centric urban sensing systems. Proceedings of the IEEE INFOCOM.

[15] Sun J. (2013). Privacy-preserving verifiable incentive mechanism for crowdsourcing market applications. arXiv.

[16] Li M., Li P., Guo L., Huang X. PPER: Privacy-preserving economic-robust spectrum auction in wireless networks. Proceedings of the IEEE INFOCOM.

[17] Sun J., Ma H. Privacy-preserving verifiable incentive mechanism for online crowdsourcing markets. Proceedings of the IEEE 23rd International Conference on Computer Communication and Networks (ICCCN).

[18] Agrawal R., Kiernan J., Srikant R., Xu Y. Order preserving encryption for numeric data. Proceedings of the 2004 ACM SIGMOD International Conference on Management of Data.

[19] Zhao D., Li X.Y., Ma H. How to crowdsource tasks truthfully without sacrificing utility: Online incentive mechanisms with budget constraint. Proceedings of the IEEE INFOCOM.

[20] Gao H., Liu C.H., Wang W., Zhao J., Song Z., Su X., Crowcroft J., Leung K.K. (2015). A survey of incentive mechanisms for participatory sensing. IEEE Commun. Surv. Tutor..

[21] Jin H., Su L., Ding B., Nahrstedt K., Borisov N. Enabling privacy-preserving incentives for mobile crowd sensing systems. Proceedings of the 2016 IEEE 36th International Conference on Distributed Computing Systems.

[22] Jin H., Su L., Xiao H., Nahrstedt K. Inception: Incentivizing privacy-preserving data aggregation for mobile crowd sensing systems. Proceedings of the 17th International Symposium on Mobile Ad Hoc Networking and Computing.

[23] Christin D. (2016). Privacy in mobile participatory sensing: Current trends and future challenges. J. Syst. Softw..

[24] Javanmardi S., Shojafar M., Shariatmadari S., Ahrabi S.S. (2014). Fr trust: A fuzzy reputation-based model for trust management in semantic P2P grids. Int. J. Grid Util. Comput..

[25] Baccarelli E., Cordeschi N., Mei A., Panella M., Shojafar M., Stefa J. (2016). Energy-efficient dynamic traffic offloading and reconfiguration of networked data centers for big data stream mobile computing: Review, challenges, and a case study. IEEE Netw..

[26] Sweeney L. (2002). k-anonymity: A model for protecting privacy. Int. J. Uncertain. Fuzziness Knowl. Based Syst..

[27] Kalnis P., Ghinita G., Mouratidis K., Papadias D. (2007). Preventing location-based identity inference in anonymous spatial queries. IEEE Trans. Knowl. Data Eng..

[28] Gedik B., Liu L. (2008). Protecting location privacy with personalized k-anonymity: Architecture and algorithms. IEEE Trans. Mob. Comput..

[29] Shin M., Cornelius C., Kapadia A., Triandopoulos N., Kotz D. (2015). Location privacy for mobile crowd sensing through population mapping. Sensors.

[30] Shilton K., Burke J.A., Estrin D., Hansen M., Srivastava M. (2008). Participatory Privacy in Urban Sensing. http://escholarship.org/uc/item/90j149pp#page-5.

[31] Shin M., Cornelius C., Peebles D., Kapadia A., Kotz D., Triandopoulos N. (2011). AnonySense: A system for anonymous opportunistic sensing. Pervasive Mob. Comput..

[32] De Cristofaro E., Soriente C. Short paper: PEPSI—Privacy-enhanced participatory sensing infrastructure. Proceedings of the ACM on Wireless Network Security.

[33] Rabin M.O. (1981). How to Exchange Secrets with Oblivious Transfer. https://eprint.iacr.org/2005/187.pdf.

[34] Zhang B., Liu C.H., Lu J., Song Z., Ren Z., Ma J., Wang W. (2016). Privacy-preserving QoI-aware participant coordination for mobile crowdsourcing. Comput. Netw..

[35] Naor M., Pinkas B., Sumner R. Privacy preserving auctions and mechanism design. Proceedings of the 1st ACM Conference on Electronic Commerce.

[36] Juels A., Szydlo M. (2003). A two-server, sealed-bid auction protocol. Proceedings of the Springer Financial Cryptography.

[37] Jaimes L.G., Vergara-Laurens I.J., Raij A. (2015). A survey of incentive techniques for mobile crowd sensing. IEEE Int. Things J..

[38] He D., Chan S., Guizani M. (2015). User privacy and data trustworthiness in mobile crowd sensing. IEEE Wirel. Commun..

[39] Zhao M., Zhou W., Gurney A.J., Haeberlen A., Sherr M., Loo B.T. Private and verifiable interdomain routing decisions. Proceedings of the ACM SIGCOMM 2012 Conference on Applications, Technologies, Architectures, and Protocols for Computer Communication.

[40] Wu F., Huang Q., Tao Y., Chen G. (2015). Towards privacy preservation in strategy-proof spectrum auction mechanisms for noncooperative wireless networks. IEEE/ACM Trans. Netw..

[41] Tran-Thanh L., Stein S., Rogers A., Jennings N.R. Efficient crowdsourcing of unknown experts using multi-armed bandits. Proceedings of the European Conference on Artificial Intelligence.

[42] Boneh D., Naor M. Timed Commitments. Proceedings of the CRYPTO 2000.

[43] Rabin M.O., Thorpe C. (2006). Time-Lapse Cryptography.

[44] Tzeng W.G. (2004). Efficient 1-out-of-n oblivious transfer schemes with universally usable parameters. IEEE Trans. Comput..

[45] Camenisch J.L., Piveteau J.M., Stadler M.A. Blind signatures based on the discrete logarithm problem. Proceedings of the Springer Advances in Cryptology—EUROCRYPT.

[46] Paillier P. Public-key cryptosystems based on composite degree residuosity classes. Proceedings of the Springer Advances in Cryptology—EUROCRYPT.

[47] Frikken K. Privacy-preserving set union. Proceedings of the Applied Cryptography and Network Security.

